# Microbial industrial enzymes: An annotated selection of World Wide Web sites relevant to the topics in microbial biotechnology

**DOI:** 10.1111/1751-7915.13469

**Published:** 2019-08-05

**Authors:** Lawrence P. Wackett

**Affiliations:** ^1^ Department of Biochemistry, Molecular Biology and Biophysics, BioTechnology Institute University of Minnesota St Paul MN 55108 USA

## Industrial enzymes and biocatalysis

https://link.springer.com/chapter/10.1007/978-3-319-52287-6_28

This review article in an e‐book provides an excellent overview to the field of industrial enzymes. In addition to the current commonly used enzymes, it contains information of emerging enzymes of interest.

## Enzymes at work

http://www.novozymes.com/-/media/Project/Novozymes/.../Enzymes_at_work.pdf

This web brochure produced by Novozymes provides many examples of industrial enzymes, their origins and applications.

## Enzymes in detergents

http://www1.lsbu.ac.uk/water/enztech/detergent.html

This web article is a little dated but it nevertheless provides a good overview of how enzymes are used on washing formulations.

## Phytase

https://www.aasv.org/shap/issues/v18n2/v18n2p90.html

Phytase is an enzyme that releases phosphate from phytate, a hexaphosphate ester of inositol. Because much phosphate in grain and seeds is stored in phytate, phytase enzyme is added to animal feed to help release phosphate from the feed.

## Lipase industrial applications

https://www.ocl-journal.org/articles/ocl/pdf/2017/04/ocl170015.pdf

Lipases, mostly obtained from fungi, is used extensively in industry. Major uses are in detergents and in food oil processing.

## Subtilisin: HERA Project

https://www.heraproject.com/files/22-F-07_PROTEASE_HERA_Final%20Edition%20(unsecured%20-%20PDFA-1b).pdf

Subtilisin is a protease, originally obtained from strains of *Bacillus*. It is used in laundry, dishwashing and contact lens cleaning applications. The current document contains much information relevant to the industrial use of subtilisin.

## Chymosin

https://www.chemistryworld.com/podcasts/chymosin/3007682.article

Chymosin is an important enzyme used in the conversion of milk to cheese. It was originally obtained from the digestive tracts of young ruminant animals. More recently, it is produced in recombinant form by microbial fermentation processes.

## Cellulases from *Trichoderma reesei*

https://microbialcellfactories.biomedcentral.com/articles/10.1186/s12934-016-0507-6

Cellulases are important industrial enzymes in biomass conversion. A major organism long studied for it enzymes for degrading cellulose is the fungus *Trichoderma reesei*.

## Microbial alpha‐amylase in industry

https://www.ncbi.nlm.nih.gov/pmc/articles/PMC3769773/

This review article discusses the uses of amylases, mostly in converting various food products such as starches in corn, wheat, rice, and tapioca. A very large process currently is the conversion of corn starch to glucose by amylase coupled to the isomerization of glucose to fructose.

## Xylanases and industrial applications

https://www.researchgate.net/publication/315803185_Xylanases_and_their_industrial_applications_A_review

This Research Gate website contains an abstract on xylanases and a link to the full review article on the topic.

## Penicillin acylase

https://www.ncbi.nlm.nih.gov/pmc/articles/PMC3347563/

This review article covers enzymes used in the production of semi‐synthetic beta‐lactam antibiotics. The major focus is on protein expression and engineering.

## Glucose isomerase

https://www.creative-enzymes.com/similar/glucose-isomerase_319.html

Glucose isomerase, also known as xylose isomerase, is widely used in the production of high‐fructose corn syrup.

## Pectinase

https://www.sciencedirect.com/topics/biochemistry-genetics-and-molecular-biology/pectinase

Pectinases are widely used in producing clarified fruit juices. The pectinases hydrolyze linkages in the pectic substance found in the cell walls of plants.

## Novozymes

https://www.novozymes.com/en

Novozymes is a major enzyme producing company, headquartered in Denmark.

## DuPont Industrial Biosciences

http://biosciences.dupont.com

DuPont is a very large producer of enzymes with currently 900 commercial products on the market.

## DuPont Primagreen

https://www.dupont.com/products-and-services/industrial-biotechnology/industrial-enzymes-bioactives/primagreen.html

Many enzymes are used in textile processing and clothing finishing. This website addresses are group of enzymes used in those applications.

## Danisco animal nutrition

http://animalnutrition.dupont.com/productsservices/feed-enzymes/

Animal nutrition enzymes are largely derived from bacteria and aid in the animal digestion of their foods. The enzymes are principally phytase, proteases, and carbohydrate hydrolyzing enzymes.

## BASF enzymes

https://www.basf.com/global/en/products/cross-industry-solutions/enzymes.html

The German chemical company BASF made a large entry into industrial enzymes with the the purchase of the Verenium Corporation.

## Amano enzymes

https://www.amano-enzyme.com/uk/about-company/

Amano produces microbial enzymes for use in food, supplement, and medical diagnostic industries.

